# Supplementary material for: The metabolic enzyme fructose-1,6-bisphosphate aldolase acts as a transcriptional regulator in pathogenic Francisella
Source: Nat Commun. 2017 Oct 11;8:853. doi: 10.1038/s41467-017-00889-7 (PMC5636795; doi:10.1038/s41467-017-00889-7)
Supplement: Supplementary file 1 — Supplementary Information [file 41467_2017_889_MOESM1_ESM.pdf]

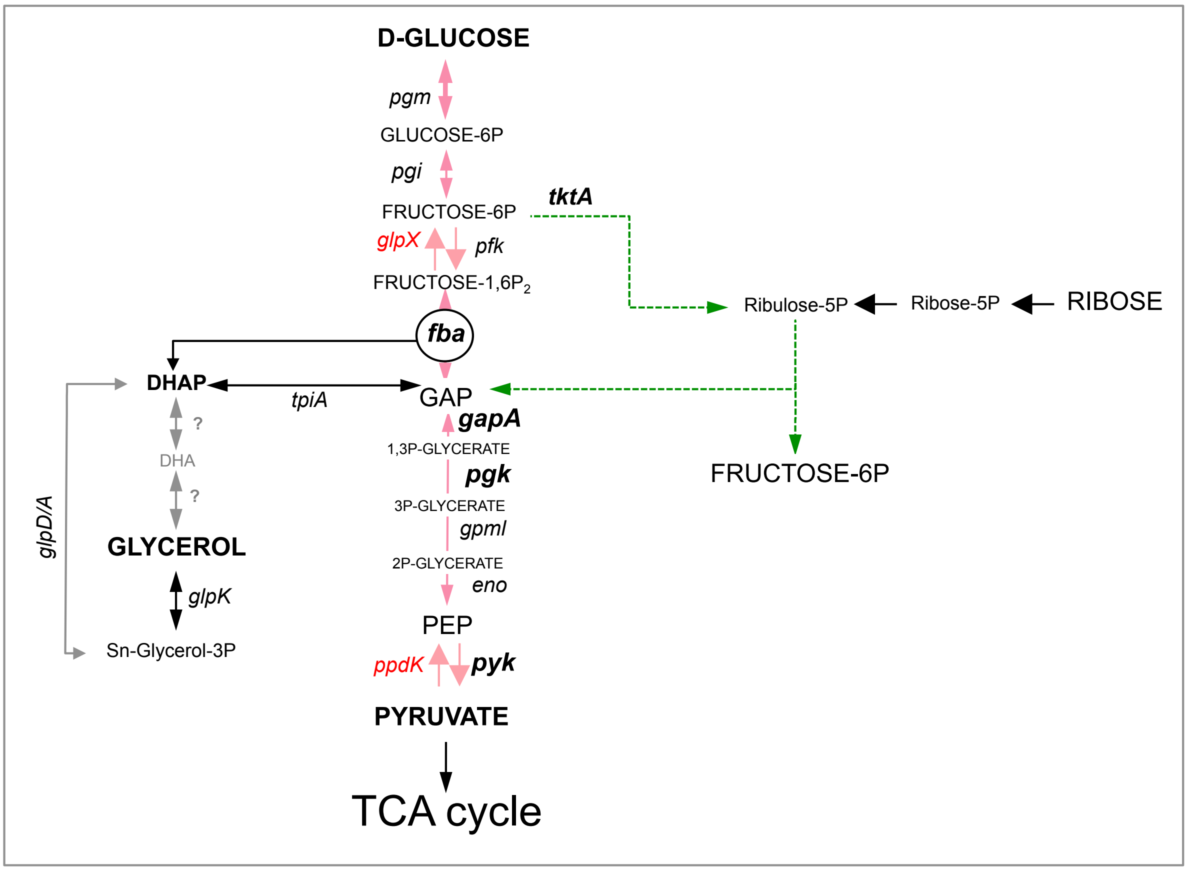

Supplementary Figure 1: Central role of FBA in glycolysis – gluconeogenesis pathways. The gene *fba* (*FTN_1329*) encodes the class II fructose 1,6-bisphosphate aldolase FBA. This enzyme catalyzes a reversible reaction that splits the 6-carbon fructose 1,6-bisphosphate, into the triose phosphates dihydroxyacetone phosphate (DHAP or Glycerone-Phosphate) and glyceraldehyde 3-phosphate (GAP). Gene names are indicted in italics: *pgm* (*FTN_0514*); *pgi* (*FTN_0663*); *gpml* (*FTN_0648*); *eno* (*FTN_0621*); *glpX* (*FTN_0298*); *pfk* (*FTN_1210*); (*FTN_1631*); *glpD* (*FTN_1584*); *glpK* (*FTN_1585*). Ppdk and FBPase (*glpX*-encoded) (in red) represent the only two enzymatic steps specifically acting in the gluconeogenic direction. The dotted green arrows indicate the non-oxidative part of the Pentose Phosphate Pathway.

a

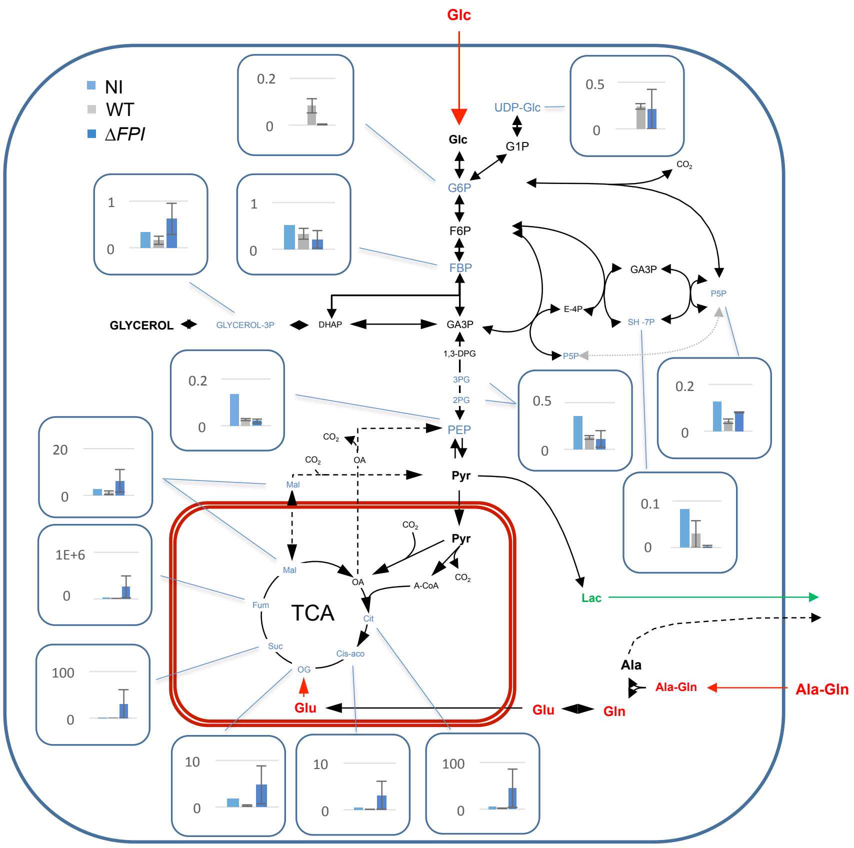
Glc
NI
WT
ΔFPI
UDP-Glc
G1P
G6P
F6P
FBP
GA3P
E-4P
SH -7P
P5P
GLYCEROL
GLYCEROL-3P
DHAP
1,3-DPG
3PG
2PG
PEP
Pyr
OA
Mal
TCA
Fum
Suc
OG
Cit
Cis-aco
A-CoA
Lac
Ala
Ala-Gln
Glu
Gln

b

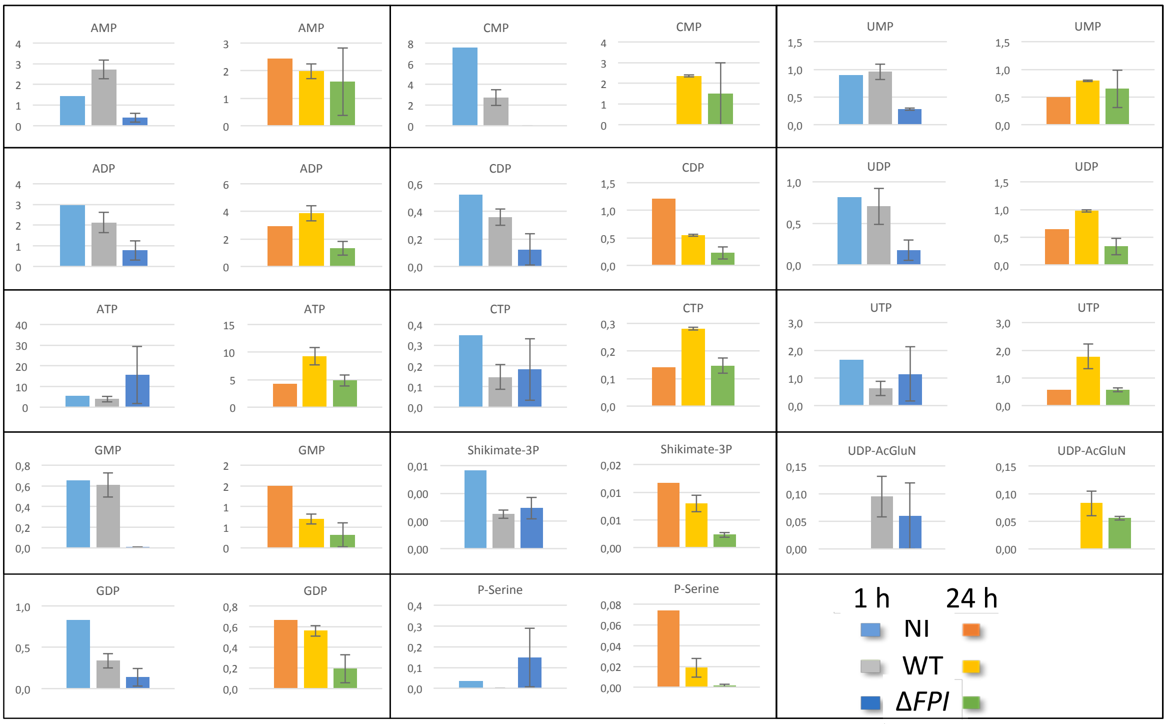
AMP
CMP
UMP
ADP
CDP
UDP
ATP
CTP
UTP
GMP
Shikimate-3P
UDP-AcGluN
GDP
P-Serine
1 h
24 h
NI
WT
ΔFPI

Supplementary Figure 2: Supplementary metabolomics data. Related to Figure 1. (**a**) Absolute concentrations of central metabolites in intracellular cell extracts (in µmol L^{-1}) after 1 h of cultivation of BMM macrophages: non infected (NI)(n=1), or infected either with wild-type *F. novicida* (WT) (n=3) or Δ*FPI* (ΔFPI) strain (n=3). Fumarate measurements (Fum) represent MS peak area instead of absolute concentrations. (**b**) Absolute concentrations of other intracellular metabolites in cell intracellular cell extracts (in µmol L^{-1}) after 1 h and 24 h of cultivation of BMM macrophages: non infected (NI; n=1), or infected either with wild-type *F. novicida* (WT) strain (n=3) or Δ*FPI* strain (n=3).

Each sample was run in technical triplicates (mean and SD of metabolite concentrations were calculated using R 3.2.3, R Foundation for Statistical Computing, Vienna, Austria. URL http://www.R-project.org/).

```
  1 MALVSLRQLLDHAAEHGYGLPAFNVNNLEQVRAVMEAADKVNSPVILQGSAGARKYAGAS   | ftn:FTN_1329
  1 MALVSLRQLLDHAAEHGYGLPAFNVNNLEQVRAVMEAADKVNSPVILQGSAGARKYAGAS   | ftu:FTT_1365c
  1 MALVSLRQLLDHAAEHGYGLPAFNVNNLEQVRAVMEAADKVNSPVILQGSAGARKYAGAS   | ftl:FTL_1149
  1 MALVSMRQLLDHAAENSYGLPAFNVNNLEQMRAIMEAADQVNAPVIVQASAGARKYAGAP   | nma:NMA0587

 61 FIRHLVLAAIEEYPHIPVCMHQDHGTSPSVCQRSIQLGFSSVMMDGSLKSDGKTPADYEY
 61 FIRHLVLAAIEEYPHIPVCMHQDHGTSPSVCQRSIQLGFSSVMMDGSLKSDGKTPADYEY
 61 FIRHLVLAAIEEYPHIPVCMHQDHGTSPSVCQRSIQLGFSSVMMDGSLKSDGKTPADYEY
 61 FLRHLILAAVEEFPHIPVVMHQDHGASPDVCQRSIQLGFSSVMMDGSLMEDGKTPSSYEY

121 NVNVTKTVSDMAHACGVSVEGELGCLGSLETGQAGEEDGIGAEGTLSMDQLLTDPEEAAD
121 NVNVTKTVSDMAHACGVSVEGELGCLGSLETGQAGEEDGIGAEGTLSMDQLLTDPEEAAD
121 NVNVTKTVSDMAHACGVSVEGELGCLGSLETGQAGEEDGIGAEGTLSMDQLLTDPEEAAD
121 NVNATRTVVNFSHACGVSVEGEIGVLGNLETGEAGEEDGVGAVGKLSHDQMLTSVEDAVR

181 FVRRTKVDALAIAIGTSHGAYKFTKPPTGDVLSIKRVKEIHARIPDTHLVMHGSSSVPQD
181 FVRRTKVDALAIAIGTSHGAYKFTKPPTGDVLSIKRVKEIHARIPDTHLVMHGSSSVPQD
181 FVRRTKVDALAIAIGTSHGAYKFTKPPTGDVLSIKRVKEIHARIPDTHLVMHGSSSVPQD
181 FVKDTGVDALAIAVGTSHGAYKFTRPPTGDVLRIDRIKEIHQALPNTHIVMHGSSSVPQE

241 WLEVINTYGGAMGETYGVPVEEIVEAIKYGVRKINIDTDLRMAATGAIRRFLAENPAEFD
241 WLEVINTYGGAMGETYGVPVEEIVEAIKYSVRKINIDTDLRMAATGAIRRFLAENPAEFD
241 WLEVINTYGGAMGETYGVPVEEIVEAIKYSVRKINIDTDLRMAATGAIRRFLAENPAEFD
241 WLKVINEYGGNIGETYGVPVEEIVEGIKHGVRKVNIDTDLRLASTGAVRRYLAENPSDFD

301 PRKYNAVAKAAMSEICAARYEAFGSAGMASKIKPISLETMFQRYESGELDPIVK
301 PRKYNAVAKAAMSEICAARYEAFGSAGMASKIKPISLEIMFQRYESGELDPIVK
301 PRKYNAVAKAAMSEICAARYEAFGSAGMASKIKPISLEIMFQRYESGELDPIIK
301 PRKYLSKTIEAMKQICLDRYLAFGCEGQAGKIKPVSLEKMANRYAKGELNQIVK
```

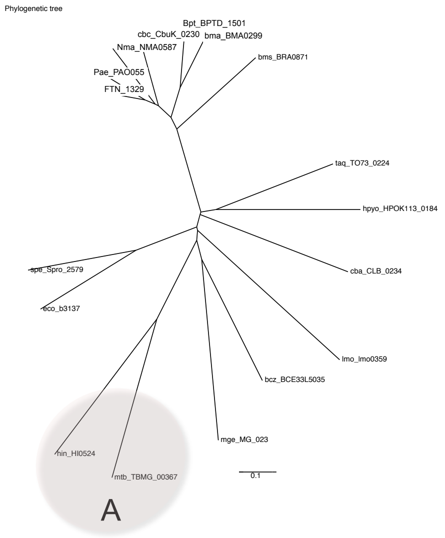

Active site
DHAP binding site
Plasminogen binding site

Supplementary Figure 3: Amino acid sequences alignment of FBA proteins. The protein FBA (FTN_1329 in *F. novicida*) shows 99.2% to 99.4% amino acid sequence identity with its orthologues in *F. tularensis* subspecies *tularensis* and *holarctica* (FTT_1365c and FTL_1149, respectively); 74.3% identity with *Neisseria meningitidis* (NMA0587) FBA II and 34.5 % with FBA from *Escherichia coli* K12 (strain MG1655: b3137). A, class IIA FBAs. For all these proteins, the Fructose 1,6-bis phosphate aldolase functional domain (Pfam) is predicted (from position 5 to 327 of the alignment, FTN numbering). Putative FBA active sites are indicated in light grey boxes. Identical position (.), gap (-).

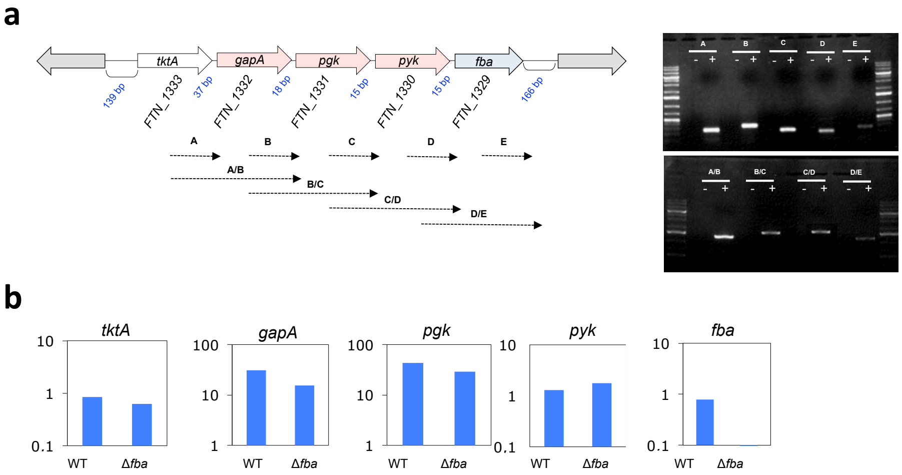

Supplementary Figure 4: The *FBA* locus of *Francisella*. (**a**) Schematic organization of the *fba* locus of *F. novicida* and transcriptional analysis. The gene *fba* (in light blue) is the last gene of a five-gene cluster, flanked by two genes in opposite orientation. Reverse transcription-PCR (RT-PCR) analyses were performed on wild-type *F. novicida* U112 strain, grown in TSB-cysteine. The dotted arrows represent the amplified region with the corresponding primer pairs. In blue, the size in base pairs (bp) of the intergenic regions. The amplified products were subjected to 1% Tris-acetate-EDTA–agarose gel electrophoresis (to the right). (+) In the presence of the reverse transcriptase (RT); and (-) in the absence of the RT. The five genes of the locus were cotranscribed 2 by 2 (reactions A, B, C, D) and 3 by 3 (reactions A/B, B/C, C/D). Together, these results strongly suggest that the *FTN_1333-1329* locus constitutes an operon. (**b**) Quantification by qRT-PCR. The expression of the five genes was further quantified by qRT-PCR in wild-type and Δ*fba* mutant strains. As expected, no *fba* transcript was detected in the Δ*fba* mutant. Othewise, *fba* inactivation had no –or very minor- impact on the expression of the other genes of the operon, confirming the lack of polar effect of the Δ*fba* deletion.

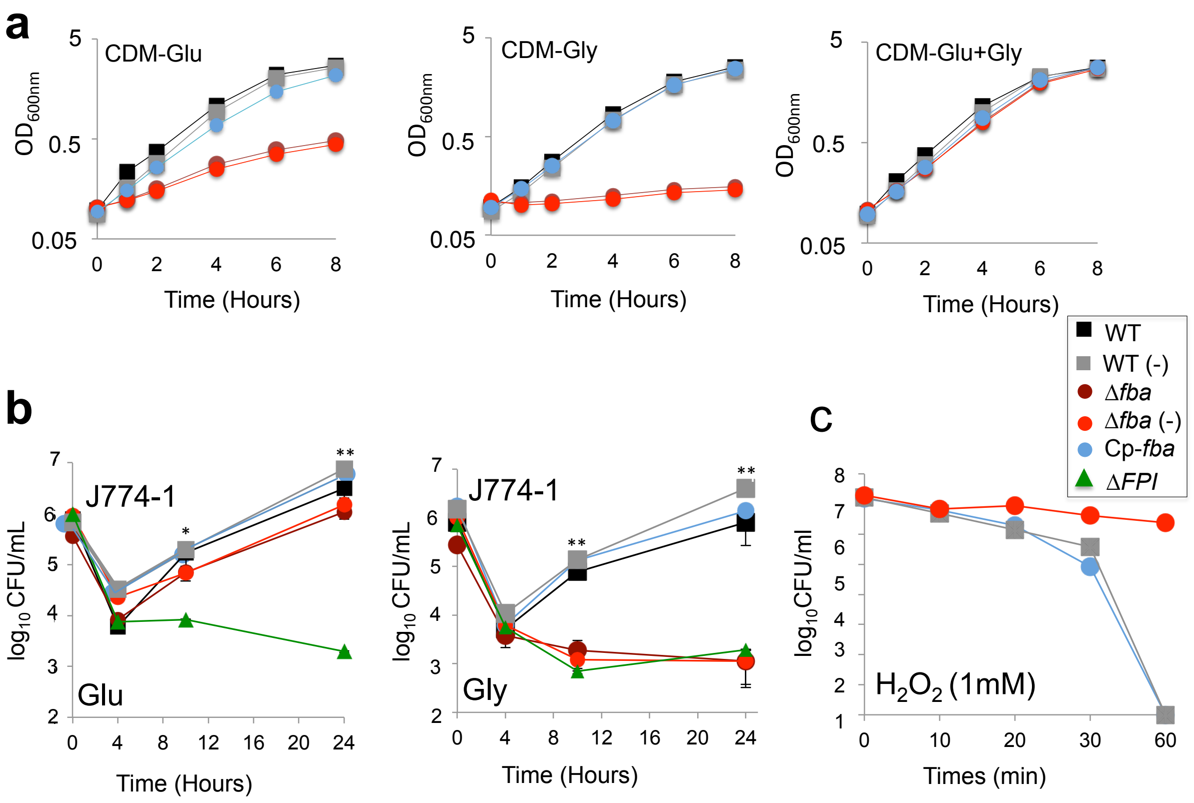

Supplementary Figure 5: The presence of the empty vector pKK214 (in wild-type or Δ*fba* mutant strains) does not affect growth in broth and in J774-1. (**a**) Wild-type *F. novicida* (WT), wild-type *F. novicida* carrying the empty vector pKK214 [WT (-)]; its isogenic Δ*fba* mutant (Δ*fba*); Δ*fba* mutant carrying the empty vector pKK214 [Δ*fba* (-)]; and *fba* complemented strain (Cp-*fba*); were grown in Chemically Defined Medium (CDM) lacking glucose and supplemented with different carbon source at a final concentration of 25 mM: from lefty to right, Glucose (Glu); Glycerol (Gly); Glucose+Glycerol (Glu+Gly).(**b**) Intracellular bacterial multiplication of wild-type *F. novicida*, carrying the empty vector pKK214 [WT (-)] or not (WT); isogenic Δ*fba* mutant carrying the empty vector pKK214 [Δ*fba* (-)] or not (Δ*fba*); complemented *fba* strain (Cp-*fba*), and Δ*FPI* mutant, was monitored during 24 h in J774A.1 macrophage cells. DMEM was supplemented either with glucose (Glu) or glycerol (Gly), at a final concentration of 5 mM. Results are shown as the average of $\log_{10}$ cfu mL−1 ± standard deviation. Each experiment was performed in triplicate. **, *p*<0.01; *, *p*<0.05 (as determined by the Student's *t*-test). (**c**) After an overnight culture in CDM supplemented with glucose and glycerol, bacteria were diluted in PBS and were subjected to oxidative stress (1 mM H_2O_2). Wild-type *F. novicida*, carrying the empty vector pKK214 [WT (-)] or not (WT); isogenic Δ*fba* mutant carrying the empty vector pKK214 [Δ*fba* (-)] or not (Δ*fba*); complemented *fba* strain (Cp-*fba*),

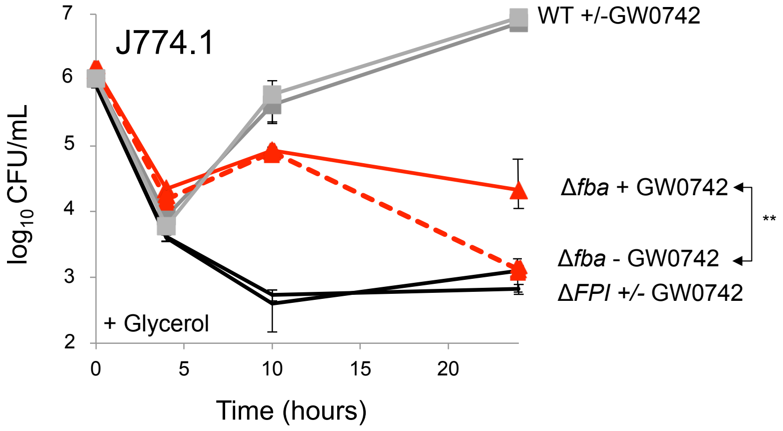

Supplementary Figure 6: Effect of the PPAR∂ agonist GW0742 on intracellular multiplication. J774.1 macrophages were infected in DMEM-glycerol with 100 MOI of wild-type *F. novicida* (WT), *Δfba* mutant, complemented strain (Δ*fba*-Cp *fba*), and Δ*FPI* mutant (Δ*FPI*) in the presence (solid red line) or absence of GW0742 (dotted red line) for 24 h. Results are shown as the average of $\log_{10}$ cfu mL−1 ± standard deviation. Each experiment was performed in triplicate. Mean and sd of triplicate wells of one typical experiment are shown. **, $p<0.05$ (as determined by two-tailed-unpaired Student's *t*-test).

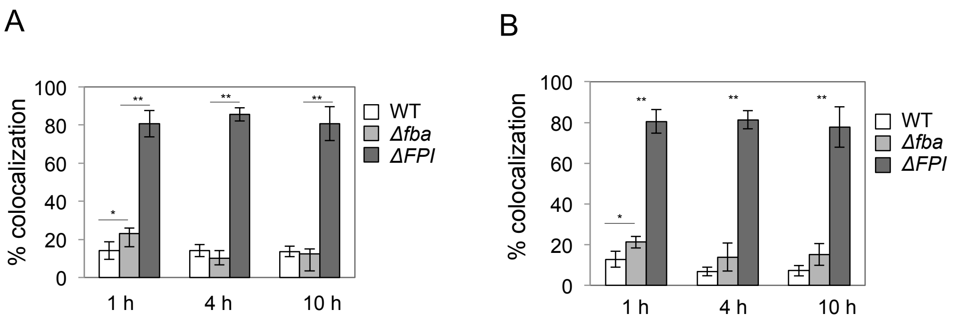

Supplementary Figure 7. Subcellular localization. J774.1 macrophages were infected for 30 min with wild-type *F. novicida* (WT), Δ*fba* or Δ*FPI* strains and their colocalization with the phagosomal marker LAMP1 was observed by confocal microscopy 1 h, 4 h and 10 h, after beginning of the experiment. Quantification of bacteria/phagosome colocalization (**a**) in glycerol-grown J774.1. macrophages; and (**b**) in glucose-grown J774.1 macrophages. Mean and sd of triplicate wells. *p<0.05; **p<0.001 (determined by two-tailed-unpaired student's *t*-test).

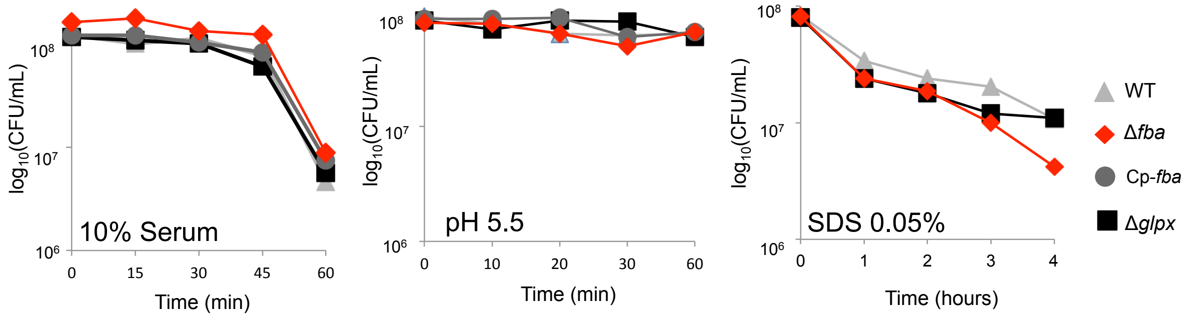

Supplementary Figure 8. Stress sensitivity assays. Related to Figure 4. Survival test were performed on wild-type *F. novicida* (WT), Δ*fba* mutant, complemented strain (Δ*fba-Cp fba*) and Δ*glpX* mutant strain (negative control). After growth on TSB-cysteine at the stationnary phase, the bacteria were diluted in PBS1x and subjected to: serum stress (10%), acidic stress (pH 5.5) or SDS stress (0,05%). Bacteria were plated on chocolate agar plates at different times and viable bacteria were monitored 2 days after. Experiments were realized twice. Data are the average cfu mL^{-1} of two values. In the three stresses tested, the survival of the four strains was unaffected.

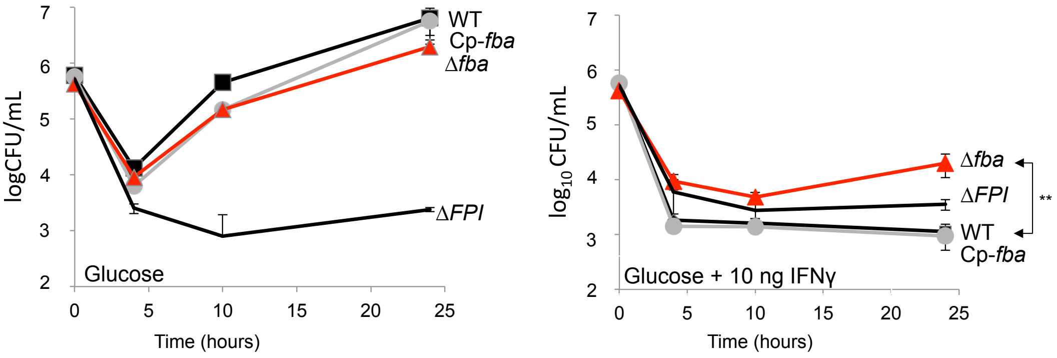

Supplementary Figure 9. Intracellular bacterial survival in IFNγ-stimulated macrophages. J774.1 macrophage-like cells were infected in DMEM-glucose with 100 MOI of wild-type *F. novicida* (WT), *Δfba* mutant, complemented strain (Δ*fba*-Cp *fba*), and Δ*FPI* mutant (Δ*FPI*), in the presence or absence of IFNγ (10 ng/ml) for 24 h. Results are shown as the average of log10 cfu mL^{-1} ± standard deviation. Each experiment was performed in triplicate. **, p<0.05 (as determined by two-tailed unpaired Student's *t*-test).

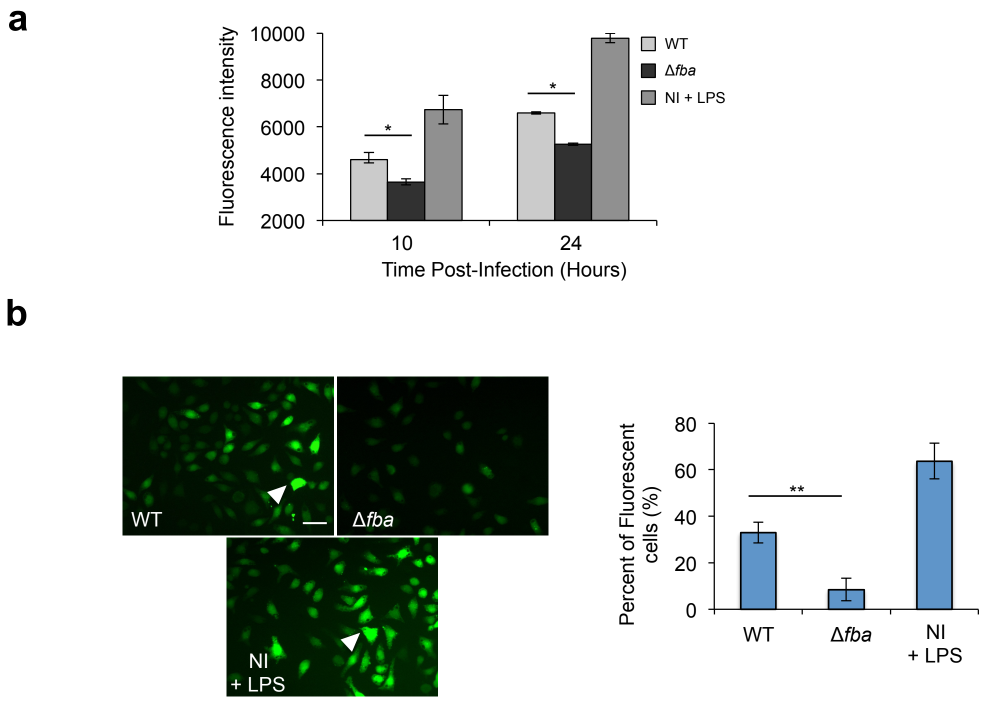

Supplementary Figure 10. ROS dosage in infected J774.1 cells. (a) ROS dosages. Generation of ROS was measured by the H2DCFDA assay in J774.1 cells infected with wild-type *F. novicida* (WT) or the Δ*fba* mutant strain, 10 h and 24 h after infection. As positive control, non-infected cells were stimulated by addition of 5µg/mL of LPS-EK Standard per wells (4.10^4 cells). Results, normalized to the protein concentration in each well, are expressed per mg of total protein. The assay was repeated twice. The histogram is representative of one typical experiment. Mean and sd of triplicate wells are shown. *, p<0.01 (as determined by two-tailed unpaired Student's *t*-test). **(b) Fluorescence microscopy**. Left panel: DCFDA levels were also visualized using fluorescence microscopy. J774.1 cells were infected with wild-type (1), Δ*fba* (2) or LPS-stimulated NI cells as positive control (3). White arrowheads indicate increased DCFDA levels. Scale bar is 50 mm. Images represent fluorescence after 1 h of DCFDA treatment. Typical fields were chosen for illustration. Right panel: Quantification of the percentage of fluorescent J774.1 cells. At least 250 cells per experiment were scored for DCFDA labeling after DCFDA treatment. *, p<0.01; **, p<0.005 (as determined by two-tailed unpaired Student's *t*-test). Scale bar at the bottom right correspond to 50 µM.

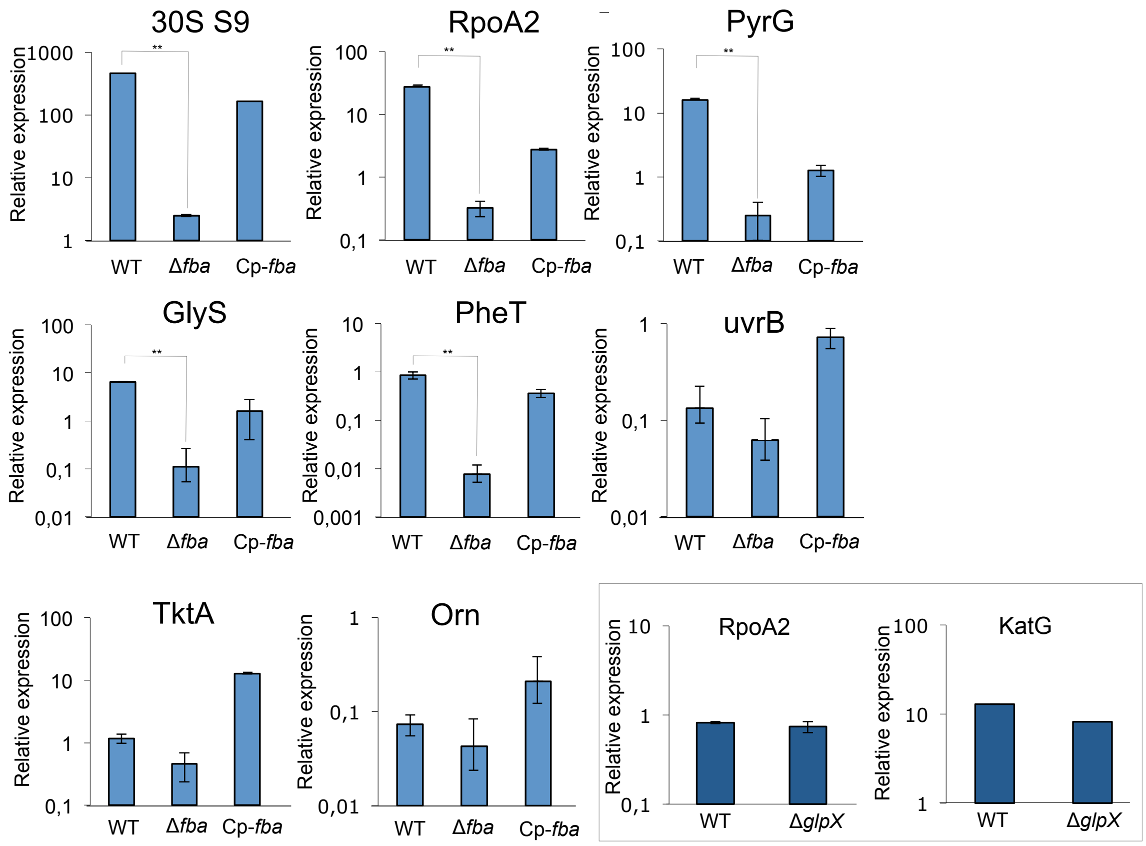

Supplementary Figure 11. qRT/PCR analyses in wild-type *F. novicida* (WT), Δ*fba* mutant and *fba*-complemented (Cp-*fba*) strains. The qRT/PCR values were normalized to the inputs and an *uvrD* (Helicase) coding region internal control. The results are expressed as relative expression of the detected fragments. Boxed to the bottom righ, expression of *rpoA* and *katG* in a Δ*glpX* mutant compared to wild-type *F. novicida*. mean and sd of triplicate of the three independent experiments are shown.*p<0.001 (determined by two-tailed unpaired student's *t*-test).

Strain	Relevant Genotype	Antibiotic resistance[a]	Relevant Features	Ref[b].
***F. novicida* U112**				
WT			*F. tularensis* subsp. *novicida* strain U112	1
U112 Δ*fba*	Δ*fba*::pGro-*nptII*	Km	Replacement of *fba* by *nptII* under control of pGro promoter	2
Δ*fba-Cp fba*$_p$	Δ*fba*::pGro-*nptII* pKK214::pGro-*fba*	Km,Tet	U112 Δ*fba* transformed with pKK214 vector containing *fba* under control of pGro promoter	2
U112-*Cp fba*	U112 pKK214::pGro-*fba*	Tet	Replacement of *pfk* by *nptII* under control of pGro promoter	2
Δ*fba* /cpFBA-HA	Δ*fba*::pGro-*nptII* pKK214::pGro-*fba* *His tag*	Km, Tet	U112 Δ*fba* transformed with pKK214 vector containing a His-tagged *fba* under control of pGro promoter	2
E. coli				
E. coli TOP10			Chemically competent cell used for routine cloning	3
Ec pKK	pKK214	Tet	*Escherichia coli* TOP10 transformed with pKK214 vector	1
Ec pKK-*fba*$_{cp}$	pKK214::pGro-*fba*	Km, Tet	*Escherichia coli* TOP10 transformed with pKK214 vector containing *fba* under control of pGro promoter	2

[a] Km: Kanamycin (10 µg.mL^{-1}), Tet: Tetracyclin (5 µg.mL^{-1})
[b] [1] lab collection, [2] this study, [3] Life Technology

Supplementary Table 1. Strains and plasmids

Primer code	Name	Sequence (5'-3')[a]	Relevant Features
p1	*fba* upstream FW	CGCGTCGACGGTAGGTAAATACCCTG	Construction of the *F. novicida* U112 Δ*fba* mutant (Δ*FTN_1329*)
p2	*fba* upstream(spl_K7) RV	[illegible]ATAAAAGGAGATAATAATAATGTTGTA GGATTA	
p5	*fba* downstream(spl_K7) FW	GAGTTCTTCTGAGGTGAGTTAGATCCA ATTG	
p6	*fba* downstream RV	CGCGTCGACGTGCTTGCTGATCTGTTG C	
p3	*pGro* FW	TTGTATGGATTAGTCGAGC	Amplification of the Km^r cassette
	pGro(spl_*nptII*) RV	TTCAATCATAACAATCTTACTCCTTTGTTA AAT	
	nptII(spl_*pGro*) FW	AAGATTGTTATGATTGAACAAGATGGATT G	
p4	*nptII* RV	TCAGAAGAACTCGTCAAGAAGGCG	
	pGro[SmaI] FW	GCCCCGGGTTGTATGGATTAGTCGAGC	Amplification of pGro promoter *from F. novicida* strain U112 used for functional complementation
	pGro RV	AACAATCTTACTCCTTTGTTAAATTAAG	
	fba FW	TGAGCTTTTTAGCTCGACTAATCCATACAA AAGATAAAAGGAGATAATAATAATGGCTT T	Amplification of *fba* gene from *F. novicida* strain U112 used for functional complementation
	fba[PstI] RV	CTACTGCAGTTATTTAACAATTGGATCTA	
	fba FW	ATGAGACGAGAACTTGCCATCG	Amplification of *fba* His tagged gene for fusion protein
	Fba HA[PstI] RV	CTACTGCAGTTAGTGGTGATGGTGATG ATGTTTAACAATTGGATCTAACTCAC	
	RT-PCR *tktA* Fw	CAGTAATAAAAGATGATATCCCGGCA G	Amplification of *tktA* gene for RT-PCR
	RT-PCR *tktA* Rv	TACCGAAACCATTAATTGCAACTCTC	
	RT-PCR *gapA* Fw	CATATCAGCGGCTTCTTGTACAACT	Amplification of *gapA* gene for RT-PCR
	RT-PCR *gapA* Rv	CCCATCTAACTTACCAGCAAGTTAAG	
	RT-PCR *pgk* Fw	AAGCCATGTAATCCAACACTCAGA	Amplification of *pgk* gene for RT-PCR
	RT-PCR *pgk* Rv	GCCTATAATTCTGCTGCCATACC	
	RT-PCR *pyk* Fw	GGTACCACACCTCTAAATAATGTCATC GCA	Amplification of *pyk* gene for RT-PCR
	RT-PCR *pyk* Rv	TACCCTGTTGAAACGGTTTCTGCAAT	
	RT-PCR *fba* Fw	GGTGTACCTGTAGAGGAAATCGTT	Amplification of *fba* gene for RT-PCR
	RT-PCR *fba* Rv	AACTCACCACTCTCATAACGTTGA	
	katG Fw	CCGACTATAGAGGTGGCTCAAATG	Amplification of *katG* gene for qPCR
	katG Rv	ACACCCACATTACCACCTAGCAC	
	fba Fw	GGTGTACCTGTAGAGGAAATCGTT	Amplification of *fba* gene for qPCR
	fba Rv	GGTGTACCTGTAGAGGAAATCGTT	

rpoA2 Fw	GGCGTAGGCTTTAGACCTGCA	Amplification of *rpoA2* gene for qPCR
rpoA2 Rv	GTAGCTGCTAGTCTTAAAGCTTCCTCG	
p-*katG* Fw	GCACTGGGACCTTTATAGATATCGCTGG	Amplification of the *katG* promoter for qPCR
p-*katG* Rv	GCATGGTGATTTCCTCGCTATAAAGTTG	
p-rpoA2 Fw	AGCCTACAGCAAGTGGTTTTGAAGC	Amplification of the *rpoA2* promoter for qPCR
p-rpoA2 Rv	CTACCCCTCTCTCTAGGGCTTCAA	
p-*hemBP* Fw	GAGTTCTTCTGAGGTGTAAATAGTCCAGTACGCGC	Amplification of the *hemBP* promoter for qPCR
p-*hemBP* Rv	TAATCCATACAACGTTCGTTCAGCAGTCACAC	
p-*fad* Fw	GCTTAGCTTGGCGTGATTTTGAACA	Amplification of the *fad* promoter for qPCR
p-*fad* Rv	GGTTTAGTGATCATAAAGACCTCCTGAAGC	
p-*uvrB* Fw	GATCACAAAGGCAAAACTCGTAAAACTCTG	Amplification of the *uvrB* promoter for qPCR
p-*uvrB* Rv	CCATTAACAAGGCTTTGTATTGCTTGAGGC	

[a] restriction sites are underlined

Supplementary Table 2. Primers
